# Long-term intelligence after high-dose radiotherapy to the primary site versus chemotherapy and whole-ventricle radiotherapy in patients with germinoma

**DOI:** 10.1007/s10147-026-02976-6

**Published:** 2026-01-30

**Authors:** Masayuki Kanamori, Yumi Sugawara, Yoshiteru Shimoda, Osamu Iizuka, Yoshinari Osada, Shota Yamashita, Ichiyo Shibahara, Rei Umezawa, Naoko Mori, Ryuta Saito, Yukihiko Sonoda, Toshihiro Kumabe, Keiichi Jingu, Shunji Mugikura, Kyoko Suzuki, Hidenori Endo

**Affiliations:** 1 Department of Neurosurgery, Tohoku University Graduate School of Medicine, 1-1 Seiryo-machi Aoba-ku, Sendai, 980-8574 Japan; 2 Division of Epidemiology, School of Public Health, Tohoku University Graduate School of Medicine, Sendai, Japan; 3 Department of Behavioral and Neurology and Cognitive Neuroscience, Tohoku University Graduate School of Medicine, Sendai, Japan; 4 Department of Neurosurgery, Kitasato University School of Medicine, Kanagawa, Japan; 5 Department of Radiology, Akita University Graduate School of Medicine, Akita, Japan; 6 Department of Neurosurgery, Nagoya University Graduate School of Medicine, Nagoya, Japan; 7 Department of Neurosurgery, Yamagata University Faculty of Medicine, Yamagata, Japan; 8 Department of Radiation Oncology, Tohoku University Graduate School of Medicine, Sendai, Japan; 9 Department of Diagnostic Radiology, Tohoku University Graduate School of Medicine, Sendai, Japan; 10 Department of Image Statistics, Tohoku Medical Megabank Organization, Tohoku University, Sendai, Japan

**Keywords:** Germinoma, Long-term follow-up, Neurocognitive function, Chemotherapy, Whole ventricular irradiation

## Abstract

**Background:**

Until 1995, patients with newly diagnosed germinoma received 40–60 Gy of radiation to the primary site with or without chemotherapy (regimen A). After 2000, treatment shifted to chemotherapy followed by 24 Gy of whole-ventricle radiation therapy (WVRT) (regimen B). This study compares long-term intelligence outcomes between the two treatment regimens.

**Methods:**

This retrospective analysis included 151 patients diagnosed with germinoma between 1983 and 2021. Intelligence was assessed using the Wechsler Adult Intelligence Scale (revised or 3rd edition) and the Wechsler Intelligence Scale for Children (3rd edition). Patient backgrounds were also collected.

**Results:**

A total of 55 and 69 patients were treated with regimens A and B, respectively. The number of patients who underwent at least one longitudinal neurocognitive assessment was 35 and 29 for regimen A and 53 and 22 for regimen B, respectively. The median interval from initial treatment to the last neurocognitive assessment was 120 months. In the longitudinal intelligence assessments, the median intervals were 58 months from treatment to the first evaluation and 83 months from the first to the final assessment. Full-Scale Intelligence Quotient (FSIQ) scores declined in regimen A but were maintained in regimen B according to analysis of covariates and generalized linear mixed model analysis.

**Conclusion:**

Chemotherapy followed by 24 Gy of WVRT appears to be associated with a smaller decline in FSIQ over a long-term follow-up.

**Supplementary Information:**

The online version contains supplementary material available at 10.1007/s10147-026-02976-6.

## Introduction

Intracranial germ cell tumors (GCTs) are rare, accounting for 2.1% of brain tumors in Japan, with germinoma being the most common subtype, comprising 41% of intracranial GCTs [1]. Germinomas respond well to radiation therapy, 40–50 Gy to the primary site, with or without craniospinal irradiation, achieving 10-year overall survival rates of 90–93% [2, 3]. However, high-dose radiation raises concerns about long-term adverse effects, particularly on performance status, neurocognitive and endocrine functions, and quality of life [3–6], especially in childhood-onset cases [3]. To reduce these risks while maintaining tumor control, treatment has shifted to platinum-based chemotherapy followed by reduced-dose whole-ventricle radiation. This approach has shown excellent tumor control, with 7- and 10-year overall survival rates of 98.8% and 97–100%, respectively [7–10]. However, the long-term impact on late adverse effects remains unclear due to limited patient numbers and short follow-up durations [5, 11–16]. In this study, by analyzing long-term outcomes in patients with germinoma treated with two regimens at our institution, we evaluated whether chemotherapy followed by reduced-dose whole-ventricle radiation better preserved intelligence over the long term compared with high-dose primary tumor site radiation therapy (PSRT), with or without craniospinal irradiation.

## Materials and methods

### Ethics

This retrospective, single-institution study was approved by the Ethics Committee of Tohoku University Hospital (2019-1-406). Patients had the option to opt out via an online platform.

### Patients

We included patients diagnosed histologically or clinically with germinoma between January 1983 and March 2021 at our department. Patients with recurrent tumors were excluded from the neurocognitive analysis. Clinical data, including age at treatment, tumor location, and treatment details, were collected from medical records as of November 2021.

### Treatments

Until 1995, patients with newly diagnosed germinoma received 40–60 Gy of radiation to the primary site with or without chemotherapy (regimen A). A two-dimensional technique with 2.0 Gy per fraction was employed to deliver PSRT. For the irradiation field, whole-brain radiation therapy (WBRT) or craniospinal irradiation (CSI) was performed, followed by boost irradiation to the enhanced lesion or local irradiation only to the enhanced lesion. Some patients receive various types of chemotherapy, including the ICE regimen (3 cycles of ifosfamide at 900 mg/m^2^, cisplatin at 20 mg/m^2^, and etoposide at 60 mg/m^2^ for 5 days, at 23-day intervals), BEP regimen (3 cycles of cisplatin at 20 mg/m^2^ and etoposide at 60 mg/m^2^ for 5 days and bleomycin at 20 mg/m^2^ at days 1, 7, and 14 at 23-day intervals), or the combination of nimustine hydrochloride and 5-FU.

Between 1996 and 1999, patients were treated with platinum-based chemotherapy alone or combined with 24 Gy of PSRT. This approach aimed to reduce radiation exposure but was insufficient in preventing recurrence. Since 2000, platinum-based chemotherapy, followed by 24 Gy in 12 fractions (2.0 Gy per fraction) of whole-ventricle radiation therapy (WVRT) without boost irradiation to the primary site using three-dimensional conformal radiation therapy (3D-CRT), has been the standard protocol (regimen B) [7, 17–19]. Two chemotherapy regimens were used: the CARE regimen (3 cycles of carboplatin 450 mg/m^2^ on day 1 and etoposide 150 mg/m^2^ on days 1–3, at 25-day intervals) and the ICE regimen. Either WBRT or CSI was administered based on lesion extent. Patients with spinal lesions or positive cytology received CSI [20], and those with basal ganglia germinoma received WBRT in regimens A and B. Before 2008, bifocal lesions were treated with WBRT or CSI in regimens A and B, and finally with 24 Gy of WVRT in regimen B. A follow-up included MRI three times per year for 5 years, twice yearly until year 10, and annually thereafter. Endocrine function was monitored and managed by pediatricians or endocrinologists after treatment.

### Neurocognitive examination

Intelligence testing began in 1990. Patients aged ≥ 16 years were assessed with the Wechsler Adult Intelligence Scale, Revised (WAIS-R) or Third Edition (WAIS-III); those < 16 years with the Wechsler Intelligence Scale for Children, Third Edition (WISC-III) [21–23].

### Statistical analysis

Chi-square test was used to compare categorical clinical characteristics between patients treated with regimens A and B. Student’s *t* test compared mean neurocognitive scores and follow-up durations, while Kruskal–Wallis test assessed differences in median values.

For the 51 patients with longitudinal assessments, analysis of covariance (ANCOVA) was conducted using the change in Full-Scale Intelligence Quotient (FSIQ), calculated as the difference between initial and most recent scores from WAIS-R, WAIS-III, or WISC-III, as the dependent variable. Covariates included age at treatment, sex, tumor location (pineal gland versus other), treatment regimen (A or B), WBRT/CSI (yes or no), and interval from initial therapy to estimation, based on prior studies [12–14], indicating their relevance to neurocognitive outcomes.

Because intelligence assessments occurred at varying times and individuals differ in baseline intelligence, we used a generalized linear mixed model with fixed effects and subject-specific random intercepts. FSIQ was the outcome variable, with treatment regimen as the primary predictor. Age at treatment, sex, tumor location, WBRT/CSI, and time from treatment to assessment were included as fixed effects. Mixed model analysis was conducted in two steps: first, using data from all patients with at least one assessment and performing sensitivity analysis on longitudinal data only; second, focusing on data collected 10–20 years post-treatment to address differences in follow-up duration between regimens A and B. Additionally, WBRT/CSI reduces the FSIQ [12]. To evaluate the effect of regimens A and B in patients treated with and without WBRT/CSI, sensitivity analysis was conducted employing a generalized mixed linear model, with stratification with WBRT/CSI.

All *p* values were two-sided, with *p* < 0.05 considered statistically significant. Analyses were conducted using SAS (version 9.4; SAS Institute Inc., Cary, NC, USA) or JMP Pro 17.0 (SAS Institute Japan Inc., Tokyo, Japan).

## Results

### Patients’ demographics

**Fig. 1:**
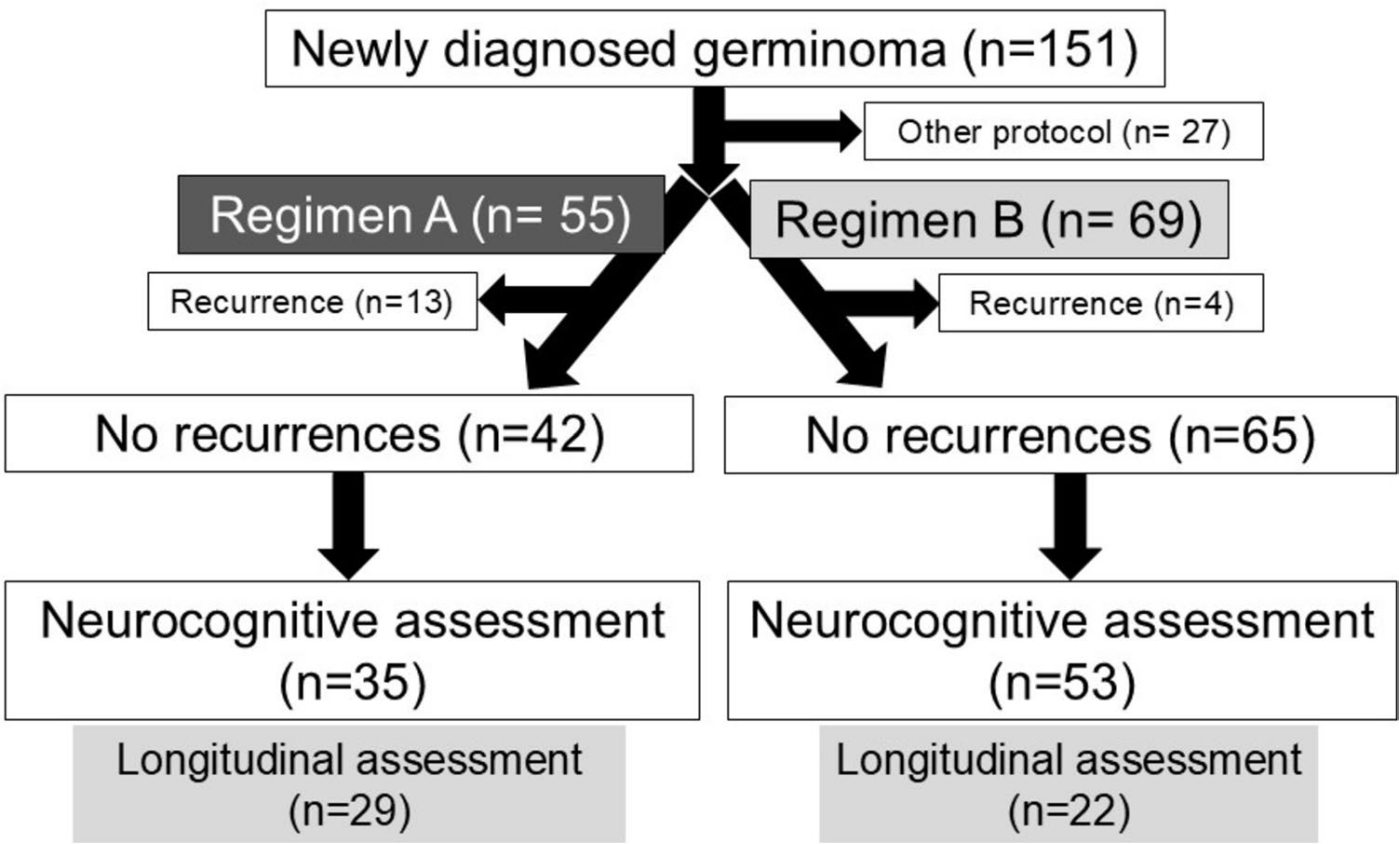
Inclusion and exclusion criteria for germinoma patients in this study

A total of 151 patients were treated during the study period. Of these, 27 patients were excluded: 9 received chemotherapy only, 12 were treated with 24 Gy of PSRT followed by platinum-based chemotherapy, and 6 underwent gamma knife treatment [24]. The number of patients who received treatment and completed at least one intelligence assessment was 55 and 35 for regimen A and 69 and 53 for regimen B (Fig. 1). The demographics of all treated patients, along with those who underwent intelligence assessments, are summarized in Table 1. In addition, the number of patients who completed longitudinal intelligence assessments was 29 for regimen A and 22 for regimen B (Fig. 1). No significant differences in age at initial treatment, sex, and tumor location were found between regimens A and B in each group. However, there was a statistically significant difference in the proportion of patients receiving chemotherapy across all groups and in those receiving WBRT/CSI among all treated patients and those with intelligence assessments. Furthermore, the radiation dose to the primary site was significantly higher in regimen A than in regimen B (Table 1), with a mean ± SD of 48.5 ± 4.9 Gy and 24.0 ± 0 Gy, respectively, in 88 patients.

**Table 1:** Patient characteristics in this study

		All patients treated (*n* = 124)			Patients evaluted with intelligence assessment (*n* = 88)			Patients evaluted with longitudinal intelligence assessment (*n* = 51)		
		Regimen A	Regimen B	*p*-value	Regimen A	Regimen B	*p*-value	Regimen A	Regimen B	*p*-value
		(*n* = 55)	(*n* = 69)		(*n* = 35)	(*n* = 53)		(*n* = 29)	(*n* = 22)	
Age at treatment (mean, SD)		16.25 ± 7.50	17.23 ± 6.86	0.45	16.20 ± 5.98	16.17 ± 5.45	0.98	16.39 ± 6.30	17.41 ± 5.01	0.50
Sex	Male	45 (81.8)	60 (87.0)	0.43	28 (80.0)	45 (84.9)	0.55	22 (75.9)	18 (81.8)	0.61
	Female	10 (18.2)	9 (13.0)		7 (20.0)	8 (15.1)		7 (24.1)	4 (18.2)	
Tumor location	Pineal gland	17 (30.9)	24 (34.8)	0.64	10 (28.6)	21 (39.6)	0.49	6 (20.7)	4 (18.2)	0.64
	Neurohypophysis	16 (29.1)	13 (18.8)		11 (31.4)	10 (18.9)		11 (37.9)	6 (27.3)	
	Bifocal lesion	16 (29.1)	23 (33.3)		9 (25.7)	16 (30.2)		7 (24.1)	9 (40.9)	
	Basal ganglia	6 (10.9)	8 (11.6)		5 (14.3)	6 (11.3)		5 (17.2)	3 (13.6)	
	Other	0	1 (1.5)							
Whole-brain radiation therapy or craiospinal radiation therapy	No	24 (43.6)	47 (68.1)	**0.0062**	11 (31.4)	34 (64.2)	**0.0027**	8 (27.6)	12 (54.6)	0.0510
	Yes	31 (56.4)	22 (21.9)		24 (68.6)	19 (35.8)		21 (72.4)	10 (45.4)	
Radiation dosage to the primary site or whole ventricle (Gy) (mean ± SPDr)	Primary site	49.3 ± 4.6	24.0 ± 0	**< 0.0001**	48.5 ± 4.9	24.0 ± 0	**< 0.0001**	48.5 ± 5.0	24.0 ± 0	**< 0.0001**
Chemotherapy	No	38 (69.1)	0	**< 0.0001**	23 (65.7)	0	**< 0.0001**	20 (69.0)	0	**< 0.0001**
	Yes	17 (30.9)	69 (100)		12 (34.3)	53 (100)		9 (31.0)	22 (100)	

Bold indicates *p*<0.05

*SD* Standard deviation, *NA* Not assessed

### Latest intelligence profile of patients treated with regimens A and B

Intelligence assessments were conducted in 88 patients. Age at the last assessment ranged from 10 to 68 years, with a mean age of 29.0 ± 11.3 years. The duration from the initial treatment to the latest intelligence assessment varied from 2 to 431 months, with a median of 120 months. Details of age at the last assessment and the interval from treatment to assessment for regimens A and B are provided in Supplementary Table 1. At the time of the most recent assessment, 5 patients were evaluated using WAIS-R and 83 with WAIS-III and WISC-III. Patients treated with regimen A were older at their final evaluation and had longer follow-up periods compared with those treated with regimen B. The mean scores for FSIQ and all index scores were below 100. Among the index scores, processing speed was relatively lower than the others.

Of the 35 patients treated with regimen A, 33 were followed for more than 10 years after treatment. In this group, the interval to the last assessment ranged from 120 to 431 months, with a median of 303 months. At the last assessment, 14 patients (42%) had an FSIQ score below 85 (−1 standard deviation).

### Comparison of intelligence between patients treated with regimens A and B using longitudinal data

We examined the effect of treatment regimens on intelligence using longitudinal data. Since a large percentage of the patients were assessed using WAIS-R at the initial assessment, we focused only on changes in FSIQ scores. Intelligence assessments were conducted two, three, and four times in 36, 14, and 1 patients, respectively. Of the 51 patients with longitudinal data, 24 were evaluated with WAIS-R at the initial assessment and with WAIS-III at the final assessment. The number of patients assessed for intelligence with WAIS-R was significantly different between the two groups (19/29 [66%] and 5/22 [22%] patients treated with regimens A and B, respectively; *p* = 0.004: Fisher’s exact test). The interval from the initial treatment to the first assessment ranged from 0 to 298 months (median 58 months), whereas the interval from the first assessment to the last ranged from 17 to 318 months (median 83 months) for all patients. Among these, 32 of the 51 patients (63%) experienced a decrease in FSIQ. A negative correlation was observed between the interval from the first to the latest assessment and the change in FSIQ score (Supplementary Fig. 1), indicating a decline of 0.33 points per year. The interval from initial assessment to last assessment was significantly different; however, compared with regimen B, regimen A demonstrated a statistically significant reduction in FSIQ in the ANCOVA (*p* = 0.0004), using the longitudinal changes in FSIQ scores from the first to the last assessment as the outcome (Table 2).

**Table 2:** Longitudinal changes of FSIQ score of 51 patients treated with regimens A and B

	Number of the patients	Interval from initial treatment to first assessment (median) (months)	Interval from initial assessment to last assessment (median) (months)	Changes of FSIQ score from initial assessment to last assessment	
				Range	Mean (SD)
Regimen A	29	0–298 (65)	30–318 (174)	− 43 to + 20	− 7.83 (13.05)
Regimen B	22	0–88 (44)	17–224 (60)	− 12 to + 16	+ 1.00 (6.70)
*p-*value*					**0.0004**

Bold indicates *p*<0.05

*FSIQ* Full-scale intelligence quotient, *SD* Standard deviation

*Analysis of covariance using age at treatment, sex (male, or female), tumor location (pineal gland, or others), radiation therapy to whole brain (yes, or no), and Interval from initial therapy to estimation as a covariate

### Evaluation of the effect of treatment regimen on FSIQ score decline using a generalized mixed linear model

**Fig. 2:**
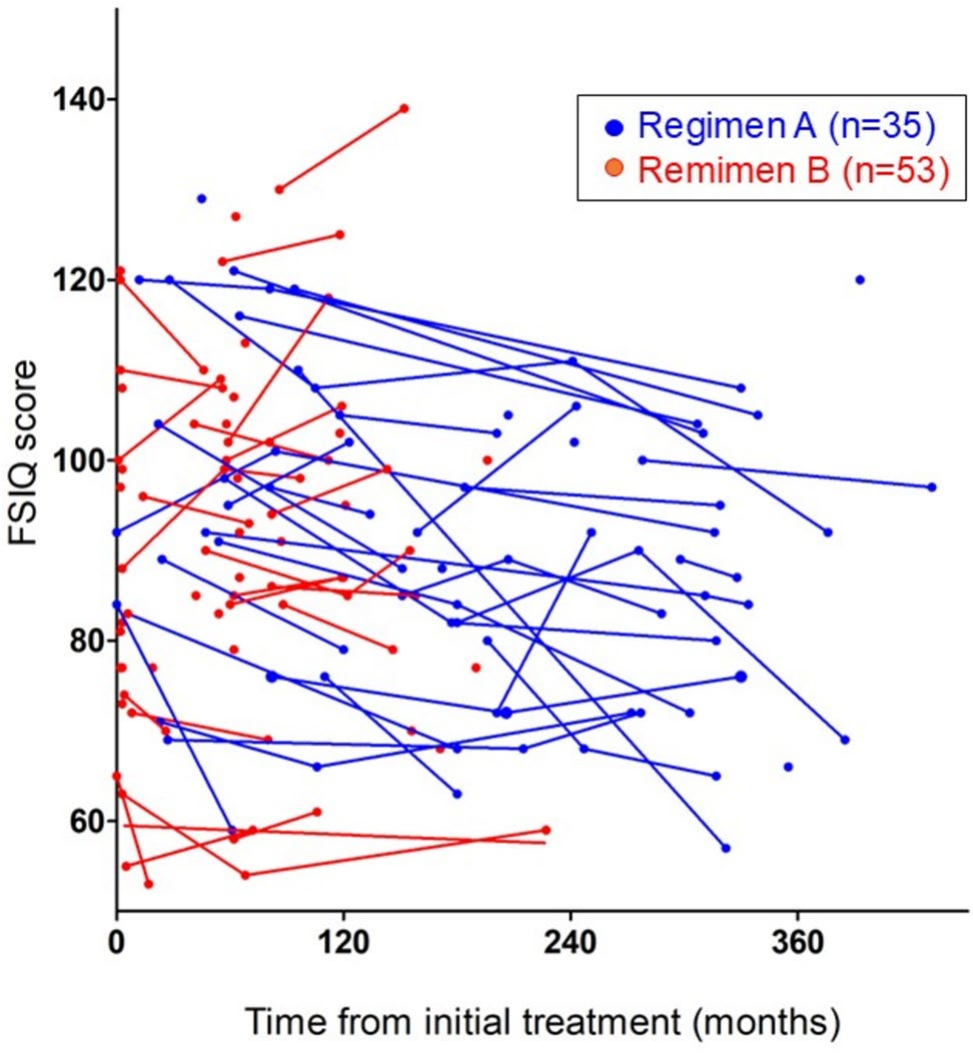
Line graph showing individual FSIQ scores and their changes over time in 88 patients evaluated. Patients treated with regimen A are shown in blue; those treated with regimen B are shown in red

The individual FSIQ scores and their changes over time are illustrated in Fig. 2. To assess the effects of the two treatment regimens on FSIQ over time, we analyzed 88 patients using a generalized linear mixed model, which accounts for individual variability in changes after treatment (Table 3). Although no statistically significant differences were found among the subgroups based on treatment regimens, sex, and WBRT/CSI, the intervals from initial treatments had a statistically significant effect on FSIQ scores (−0.56 points per year; *p* = 0.0003 for all patients). In addition, there were significant interactions between the treatment regimen and the interval from initial treatment, with a difference in the annual change in FSIQ score of 0.78 points per year for patients treated with regimen B compared to those with regimen A (*p* = 0.016) (Table 3). Sensitivity analysis of the 51 patients evaluated over time yielded similar results (Table 3). The generalized linear mixed model results indicated that FSIQ scores decline over time, with a more pronounced decrease in regimen A, while regimen B appeared to preserve the FSIQ score. In this analysis, the intervals from treatment to assessment were significantly different between the two regimens (Supplementary Table 2). This disparity could lead to misleading conclusions if the rate of intelligence decline varied over time. To address this situation, we conducted analyses focusing on data collected over similar follow-up periods of up to 10 years or 20 years after treatment. While analyzing data up to 20 years after treatment showed differences in follow-up periods, limiting the analysis to 10 years eliminated the difference between the two treatment groups (Supplementary Table 2). A generalized linear mixed model using data up to 20 years produced similar results, while the analysis up to 10 years revealed marginal effects of intervals on FSIQ scores and a significant interaction between intervals and regimens (Supplementary Table 3). These results corroborate the findings observed throughout the study period and suggest that the decline in the FSIQ scores over time with the intervals is modest in patients treated with regimen B.

**Table 3:** Effects on the FSIQ score of treatment regimens determined using mixed model regression

		All patients (*n* = 88)			Patients with longitudinal assessment (*n* = 51)		
		Estimate	SD	*p*-value	Estimate	SD	*p*-value
*Fixes effect*							
Intercept (constant)		97.43	(8.41)	**< 0.0001**	90.94	(10.64)	**< 0.0001**
Regimen B		− 8.8536	(4.9)	0.07	− 6.4906	(6.37)	0.31
Regimen A		Reference			Reference		
Interval from initial treatment (years)		− 0.56	(0.15)	**0.0003**	− 0.56	(0.14)	**0.0002**
Treatment × Interval from initial treatment (years)	Regimen B	0.78	(0.31)	**0.016**	0.85	(0.34)	**0.014**
	Regimen A	Reference			Reference		
*Moderating variables*							
Age at initial treatment		− 0.00529	(0.35)	0.99	0.15	(0.46)	0.74
Sex	Male	− 1.6756	(5.82)	0.77	− 3.9073	(6.94)	0.58
	Female	Reference			Reference		
Tumor location	Pineal gland	2.65	(4.73)	0.58	− 1.7007	(6.85)	0.81
	Others	Reference			Reference		
Radiation therapy to whole brain	Yes	4.87	(4.59)	0.29	10.98	(5.83)	0.07
	No	Reference					

Bold indicates *p*<0.05

*FSIQ* Full-scale intelligence quotient, *SD* Standard deviation

### Evaluation of the effect of the treatment regimen on FSIQ score decline employing a generalized mixed linear model, with stratification with WBRT/CSI

Supplementary Fig. 2 illustrates the individual FSIQ scores and their changes over time based on the WBI/CSI. The analysis of FSIQ scores within 20 years post-treatment identified notable interactions between the treatment regimen and the interval from initial treatment, indicating that regimen B can preserve FSIQ scores, especially in patients treated with WBRT/CSI. Among patients receiving WBRT/CSI, 21 and 20 received regimens A and B, respectively. WBRT dosage was not significantly different between the two regimens (*p* = 0.23, Wilcoxon rank test). Median and interquartile (IQR) range were 24 Gy (IQR: 24–30 Gy) and 24 Gy (IQR: 24 Gy) in regimens A and B, respectively. However, 8 patients (38%) in regimen A received 30 Gy of WBRT and CSI. In addition to the high dose to the local site (median dose: 50 Gy in regimen A and 24 Gy in regimen B; *p* < 0.0001, Wilcoxon rank test), these differences affected the decline in FSIQ in regimen A.

## Discussion

This study examined intelligence outcomes in patients who achieved tumor control following two previous treatments and were followed over an extended period. While the FSIQ score declined over time, it was preserved in regimen B as indicated by ANCOVA and the generalized linear mixed model analysis. Although the study was based on various intervals and different versions of tests used to evaluate intelligence, a major strength of our study was the evaluation of intelligence outcomes during a long-term follow-up period.

We investigated the difference between the two regimens regarding their impact on longitudinal changes in the FSIQ score, with our analyses indicating that regimen B was superior to regimen A in preserving the FSIQ score. Other factors have also been linked to late neurocognitive dysfunction, including tumor location, female sex, younger age at diagnosis, WBRT/CSI, and time from the initial treatment to the neurocognitive assessment [12, 14]. For example, Mabott et al. reported that patients treated with WBRT/CSI and younger patients at diagnosis exhibited a longitudinal decline in the FSIQ score [12]. Park et al. reported that patients with basal ganglia lesions had low FSIQ scores at baseline and that those with neurohypophyseal lesions had a longitudinal decline in the FSIQ score during follow-up [14]. We utilized ANCOVA and a generalized mixed linear model to adjust for these factors during the comparison of the two groups. Among these, the proportion of WBRT/CSI, which was significantly different between the two groups, was adjusted. Whether patients with bifocal lesions should be treated with CSI and WVRT remains controversial [25–31]; however, we revised the treatment strategy for patients with bifocal lesions. We considered bifocal lesions as disseminated disease and treated such patients with WBRT or CSI before 2007 and with WVRT thereafter. Due to the limited number of cases, we categorized tumor location into two groups—pineal lesions and lesions at other sites—based on previous reports [10, 12, 32]. Future studies with larger cohorts are warranted to enable the classification of tumors into pineal, neurohypophyseal, bifocal, and basal ganglia lesions for optimal adjustment.

Longitudinal changes in intelligence over time in patients with intracranial GCTs have been documented [5, 11–16]. Six reports were retrospective studies, while one was a prospective analysis (Supplementary Table 5). The number of cases varied from 8 to 64. The treatment strategies included high-dose (>40 Gy) PSRT plus CSI and WVRT with or without low-dose (<30 Gy) PSRT, corresponding to regimens A and B, respectively. In the longitudinal analysis, initial data were collected prior to treatment [5, 12], at the end of treatment [13, 16], or after treatment, with follow-up periods ranging from 3 to 18.5 months. The time interval from the initial assessment to the final assessment ranged from 21 to 56 months. Neurocognitive assessments focused on areas such as intelligence, memory, executive function, social behavior, and academic achievement. There remains debate regarding whether patients with germinoma maintain their neurocognitive function during long-term follow-up after various treatments. In these reports, the FSIQ score remained stable over time [11–13, 15, 16], improved in patients without neurohypophyseal lesions [14] and those with complete remission treated with 18 Gy of WVRT and a total of 30 Gy of PSRT [15], or declined in those with such lesions [14]. This study assessed the longitudinal changes in FSIQ score following high-dose PSRT or chemotherapy, followed by reduced radiation therapy covering the whole ventricle, with a long follow-up period. Three significant findings emerged. First, we showed changes in the FSIQ score over the longest intervals, both from the initial treatment to the first assessment (median 58 months) and from the first assessment to the last assessment (median 83 months). As suggested in earlier studies [15], accurately assessing neurocognitive function before treatment or shortly after can be challenging due to physical symptoms resulting from the original condition and its treatment. From this perspective, this study includes longer intervals from treatment to initial and last assessment compared to the previous study. This aspect of our study can be viewed as both a strength and a limitation when evaluating long-term intelligence outcomes. A longer interval after treatment is associated with more challenges in the interpretation of the causal relationship between intelligence outcomes and treatment regimen. Conversely, this is the first study to demonstrate a continuous decline in the FSIQ score during the chronic stage following treatment in these patients. Second, we presented long-term longitudinal changes with a median follow-up of 239 months after regimen A, which was conducted until the 1990s. During this follow-up, the FSIQ score decreased in 22 (76%) out of 29 patients, with 9 (31%) patients experiencing a decline of more than 15 points. While no studies have documented longitudinal changes in intelligence beyond 10 years, two cross-sectional studies have demonstrated performance and social status using data from a large patient cohort, in which they received the high-dose PSRT. Ogawa et al. reported on the performance status of 114 patients who received 30 Gy of CSI and WBRT and 50 Gy of PSRT [2]. During a median follow-up of 122 months, severe neurocognitive dysfunction, defined as clinically evident situations hampering social activity, was observed in only two patients. Jinguji et al. evaluated the performance and social status of 46 patients who received 26.6–26.7 Gy of CSI or WBRT along with 50 Gy of PSRT [3]. During a median follow-up of 125 months, 64% of these patients maintained a normal social life, while 19% experienced delayed neurocognitive dysfunction, defined by a low FSIQ score or clinical findings. In the present study, 42% of the patients who were treated with regimen A and followed for more than 10 years had an FSIQ score below 85. The comparison of these results with studies including data collected 10 years after treatment [2, 3] suggests that the impact of treatment on intelligence could continue or further progress over the next 10 years. Third, we compared the changes in FSIQ between patients treated with two different regimens: high-dose PSRT using a two-dimensional technique and chemotherapy, followed by low-dose WVRT. Most previous studies reported that FSIQ was preserved or improved within 5 years post-treatment in patients receiving regimen B (Supplemental Table 5) [11–16]. In contrast, our findings showed that the mean scores for FSIQ, VC, PR, WM, and PS were all below 100 in patients treated with regimen B. Nevertheless, FSIQ scores were relatively preserved with regimen B compared to those treated with regimen A.

### Limitations of this study

First, we compared longitudinal FSIQ changes between patients with significantly different treatment periods and assessment times. Cognitive scores tend to increase over time due to the Flynn effect [33], and revisions to intelligence tests have included adjustments to subtests. These changes may have led to overestimating FSIQ decline when initial and final scores were assessed with WAIS-R and WAIS-III, respectively. A high proportion of regimen A patients fell into this category, potentially biasing the longitudinal FSIQ results. Second, the longitudinal evaluation of PS and other indices, such as VC, PR, and WM, was limited by the long evaluation period, leaving the mechanism of FSIQ decline unclear. Further longitudinal studies are needed to clarify the causes. Third, regimen A used a two-dimensional radiation technique, complicating accurate estimation of radiation dose and volume. Thus, applying these findings to predict outcomes after current 3D-CRT treatment may be difficult.

## Conclusion

This study evaluated intelligence using data from patients with the longest follow-up after two treatment regimens. Although FSIQ declined over time overall, chemotherapy followed by 24 Gy of WVRT appears to be associated with a smaller decline in FSIQ over a long-term follow-up.

## Supplementary Information

Below is the link to the electronic supplementary material.

Supplementary file1 (DOCX 171 kb)

Supplementary file2 (XLSX 12 kb)

Supplementary file3 (XLSX 13 kb)

Supplementary file4 (XLSX 14 kb)

Supplementary file5 (XLSX 13 kb)

Supplementary file6 (XLSX 13 kb)

## Data Availability

No datasets were generated or analyzed during the current study.
