# Supplementary material for: Long-term intelligence after high-dose radiotherapy to the primary site versus chemotherapy and whole-ventricle radiotherapy in patients with germinoma
Source: Int J Clin Oncol. 2026 Jan 30;31(4):649–57. doi: 10.1007/s10147-026-02976-6 (PMC13018061; doi:10.1007/s10147-026-02976-6)
Supplement: Supplementary file 1 — Supplementary file1 (DOCX 171 kb) [file 10147_2026_2976_MOESM1_ESM.docx]

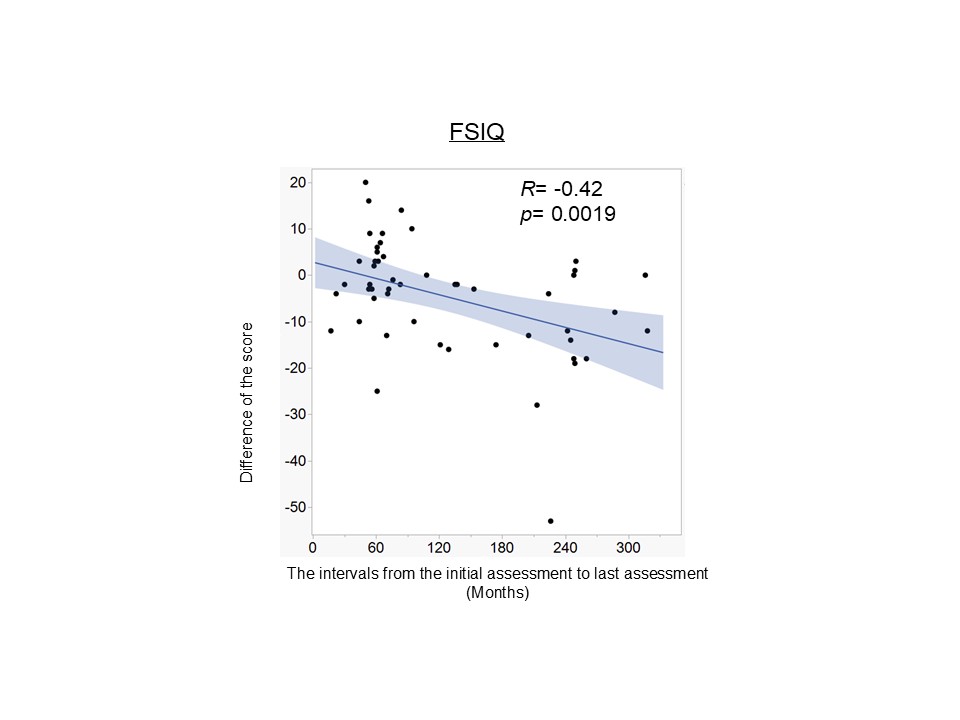


Supplementary Fig. 1. Scatter plot with linear regression and 95% confidence interval showing the relationship between the difference in Full-Scale Intelligence Quotient (FSIQ) scores and the intervals from the initial assessment to the final assessment. R = correlation coefficients. The *p*-value was determined using Pearson correlation coefficient analysis

**
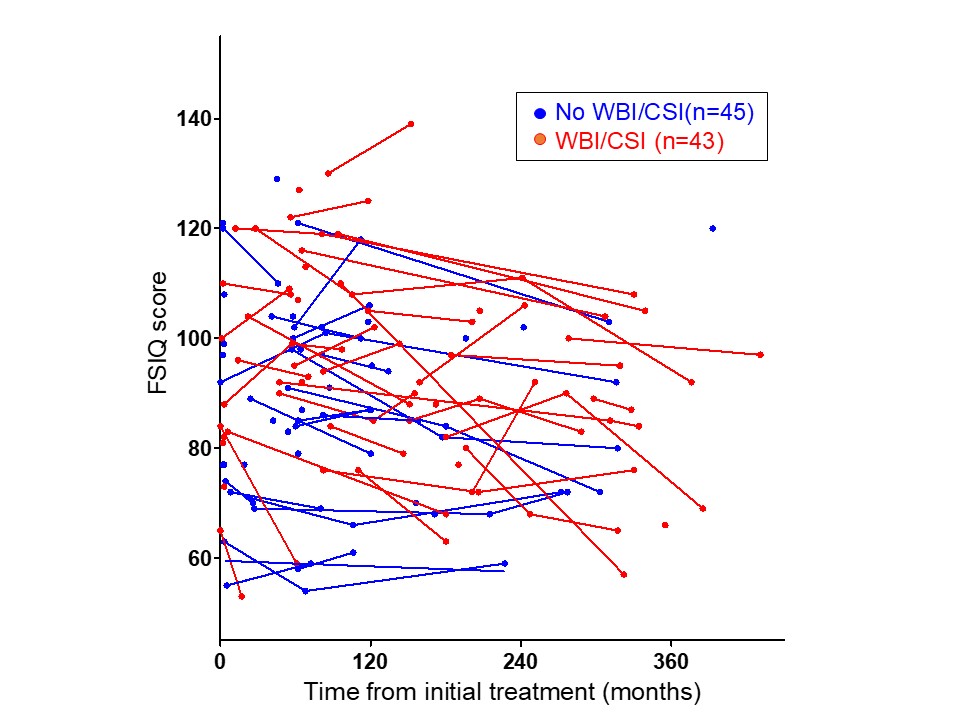
**Supplementary Fig. 2. Line graph showing individual Full-Scale Intelligence Quotient (FSIQ) scores and their changes over time in 88 patients evaluated. Patients treated without whole brain irradiation or craniospinal irradiation (WBI/CSI) are shown in blue; those treated with WBI/CSI are shown in red.
